# Recent Advances in Plasmonic Sensors

**DOI:** 10.3390/s140507959

**Published:** 2014-05-05

**Authors:** Lianming Tong, Hong Wei, Shunping Zhang, Hongxing Xu

**Affiliations:** 1 Beijing National Laboratory for Condensed Matter Physics and Institute of Physics, Chinese Academy of Sciences, Beijing 100190, China; E-Mails: lianming.tong@iphy.ac.cn (L.T.); weihong@iphy.ac.cn (H.W.); 2 Center for Nanoscience and Nanotechnology, and School of Physics and Technology, Wuhan University, Wuhan 430072, China; E-Mail: spzhang@whu.edu.cn; 3 Division of Solid State Physics/The Nanometer Structure Consortium, Lund University, Box 118, Lund S-22100, Sweden

**Keywords:** surface-enhanced Raman scattering (SERS), surface plasmon resonance (SPR) sensors, localized surface plasmon resonances (LSPRs), surface plasmon polaritons (SPPs)

## Abstract

Plasmonic sensing has been an important multidisciplinary research field and has been extensively used in detection of trace molecules in chemistry and biology. The sensing techniques are typically based on surface-enhanced spectroscopies and surface plasmon resonances (SPRs). This review article deals with some recent advances in surface-enhanced Raman scattering (SERS) sensors and SPR sensors using either localized surface plasmon resonances (LSPRs) or propagating surface plasmon polaritons (SPPs). The advances discussed herein present some improvements in SERS and SPR sensing, as well as a new type of nanowire-based SPP sensor.

## 1. Introduction

The unique optical properties of surface plasmons (SPs) have led to many important applications in multidisciplinary fields, such as chemistry, biology, materials, renewable energy and information sciences and technologies [1–5]. Plasmonic sensors, stemming from the local electromagnetic (EM) field enhancement and the ultra-sensitivity of surface plasmon resonance (SPR) to the surrounding medium, have seen prosperous growth in recent years. The sensing techniques include mainly two types: surface-enhanced spectroscopic sensors such as surface-enhanced Raman scattering (SERS) [6–8], surface-enhanced fluorescence [9,10] and surface-enhanced infrared absorption [11,12], and SPR sensors that have already led to the establishment of a number of industrial companies [13–16].

Amongst the various surface-enhanced spectroscopic sensors SERS, with its high enhancement factors and the possibility of fingerprint recognition of species, has been the most popular tool. In SERS, the enhancement is ascribed to the dominant EM contribution due to the excitation of SPR and the minor chemical contribution originating from the charge transfer effect [6,7,17–21]. In terms of EM enhancement, both the incident and the scattered electric fields are enhanced by the SERS-active substrate. As a result, the total Raman enhancement is the product of the intensity enhancements at the incident and Raman-scattered frequencies, respectively, which is approximated as the fourth power of the EM field enhancement and can reach ∼10^9^–10^10^ under optimal excitation configurations. Along with the possible chemical effect, SERS can be so sensitive that single molecules can be detected [7,22,23].

The key characteristic of a SERS-active substrate is either the nanogaps between metal nanostructures or sharp features of single nanostructures, which produce high EM field enhancement under resonant excitation [6,7,24,25]. So far, various SERS-active substrates have been prepared using either the bottom-up or the top-down process, for example, aggregates and self-assembled colloidal metal nanoparticles (NPs), nanofabricated arrays of metal NPs on substrates, metal island films and roughened electrochemical metal electrodes [8,26–33]. However, a number of drawbacks usually arise during SERS measurements on such substrates. First, the laser power on a substrate of low SERS-activity has to be high, causing potential heat damage to the sample. Second, photo-induced chemical reactions are possible, so that the intrinsic spectral information is hidden or lost and the spectral analysis becomes complicated. The reactions could occur due to either the photochemical effect by the incident laser itself or the plasmonic effects, for example, the plasmon-induced “hot” electrons generation. Third, the molecules are usually irreversibly adsorbed on the metal surface so that the SERS-substrates are not reusable, increasing the cost of SERS sensing devices. In order to overcome the above drawbacks, at least to some extent, some new SERS techniques have been demonstrated, which will be discussed below.

On the other hand, SPR sensors are based on the resonant peak shift of SPs due to the change of the refractive index of the surrounding environment [5,15]. The molecules adsorbed on the surface of metal nanostructures have different refractive index from the surrounding medium. Although the difference is tiny, it is still possible to be monitored by the shift of SPR peak positions. The amount of peak shift per refractive index unit (RIU) change, is usually defined as the sensitivity to characterize the performance of a SPR sensor is [14]. Significant efforts have been made to improve the performances of both the localized surface plasmon resonance (LSPR) sensors and the sensors based on surface plasmon polaritons (SPPs) [14,16,34,35]. To account for influence of the peak width on the sensing performance, the sensitivity is divided by the full width at half maximum, giving a unit-less figure of merit (FoM) that is a more reliable measure of the sensing performance [36]. Hence, narrow peaks are preferred for sensing purpose. The FoM values are normally less than 10 for LSPR sensors and slightly higher for SPP- based sensors [5,37].

In order to improve the FoM, a number of nanostructures and sensing techniques have been studied [38–41]. Below we will discuss that the multipolar modes of LSPR have higher FoM than that of the dipolar mode, and the Fano-resonances in plasmonic NP oligomers can also produce high FoM values for sensing.

For propagating SPPs in metal nanowires (NWs), the refractive index sensing is also possible [42,43]. The wavelength of SPP is determined by the incident wavelength and the dielectric functions of both the surroundings and the metal [44]. In a metal NW of finite length, the near-field distribution of SPP is determined by the interference of SPP modes in the NW, and can be seen visually by the quantum-dots imaging technique [45,46]. The period of the near-field distribution is a function of the dielectric constant of the surroundings. Besides, the output spectra of the NW are also modulated by the surroundings. Such features realize a new type of SPP sensor.

The scope of this review article is to present recent advances in plasmonic sensors, including some new SERS sensing techniques, LSPR sensing using multipolar modes and Fano-resonances, and the metal NWs-based SPP sensors.

## 2. SERS Sensing Techniques

Aggregates of metal NPs are typically preferred for SERS sensing due to the nanogaps wherein strong localized EM fields are confined [6,7,24]. The magnitude of the EM field is largely enhanced and is much stronger than that of a single smooth nanostructure. The field enhancement can be so high that SERS of single molecules located at the gaps can be observed [7]. Besides, the polarization of the Raman scattered light is also tunable in oligomers, which is useful for detailed study of molecule/metal interactions that would facilitate rational design of efficient SERS sensors [47,48]. The general considerations of choosing SERS substrates for sensing include large field-enhancement, stability and reusability. Herein we discuss three types of novel SERS techniques that have been recently reported: remote SERS, graphene-mediated SERS and tip-enhanced Raman scattering (TERS) in ultra-high vacuum.

### 2.1. Remote SERS

Remote SERS refers to the fact that the Raman signal is not excited by the direct illumination of the incident laser, but the laser-generated SPPs that propagate along a metal thin film or metal NW to a distal SERS-active site [49,50]. The advantages are: (1) it is particularly useful in applications where direct laser illumination should be avoided, for example, in living systems where the high laser power might cause cell destruction or induce a chemical modification of the analyte; (2) the background of the excitation laser is reduced to a great extent so that the signal to noise ratio of the Raman spectra is improved.

**Figure 1.**
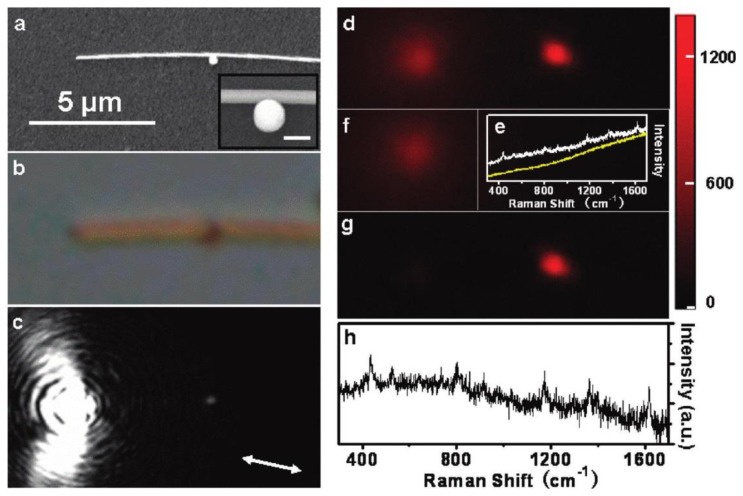
Remote SERS of MGITC excited at the particle/nanowire junction. (**a**,**b**) Scanning electron microscope (SEM) and optical images of the structure, respectively; (**c**) Optical image of SPP propagation excited by 633 nm laser; (**d**) Raman image at 436 cm^−1^; (**e**) Spectra measured at the nanojunction (white) and the excitation spot (yellow); (**f**) Background fluorescence image of the substrate; (**g**,**h**) The corresponding Raman image and SERS spectrum after background correction. Adapted with permission from [49].

Figure 1 shows an example of remote SERS measurement [49]. The Raman probe is malachite green isothiocyanate (MGITC) molecules located at the NP/NW junction (see the inset in Figure 1a). SPPs are excited by focusing the 633 nm laser at the left terminal of the NW, propagate toward the right terminal, and are partially scattered out at the NP/NW junction (Figure 1c). An enhanced EM field is thus induced due to the near-field coupling between the NP and the NW [51], and results in enhanced Raman signals as shown by the Raman image at 436 cm^−1^ in Figure 1d. After subtraction of the fluorescence background from the substrate at the excitation site (Figure 1f), the SERS spot is clearly seen in Figure 1g, which coincides with the location of the NP/NW junction. Figure 1e shows the typical SERS spectrum of MGITC at the junction and the corresponding fluorescence background, with the background-corrected spectrum shown in Figure 1h. Note that the 10^−11^ mol/L concentration of MGITC guarantees that the number of molecule in the NP/NW junction is less than one, so single molecule sensitivity is expected in this observation.

### 2.2. Graphene-Mediated SERS

Roughness on nanostructures is a necessary feature for SERS sensors. Although it has been shown that SERS on flat surfaces is possible, for example, using SPPs in a metal film, the weak field enhancement limits its practical application as SERS sensors [19,52]. Recently, Xu *et al.*, have demonstrated that graphene-mediated SERS (G-SERS), which uses the flat surface of graphene lying on gold nanoclusters to detect molecules, can provide high enhancement factors, and more importantly, clean vibrational information of the analytes free from metal-molecule interactions and high stability against photo-induced damages due to the isolation of the molecules from metal surface by the graphene layer [53].

**Figure 2.**
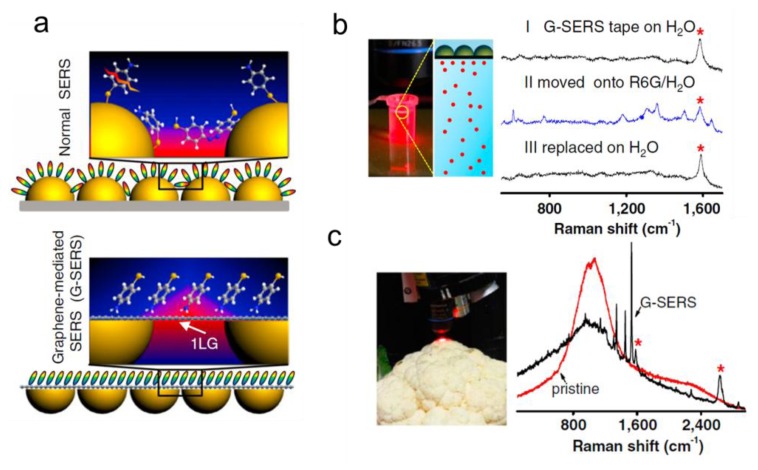
(**a**) The schemes of normal SERS and G-SERS; (**b**) Real time and reversible G-SERS characterization of R6G in a 1 × 10^−5^ M aqueous solution; (**c**) Pristine and G-SERS spectra of a cauliflower surface with adsorbed CuPc (soaked in 1 × 10^−5^ M CuPc in ethanol for 10 min). “*” marks the enhanced G-band and G'-band features of one-layer graphene. Adapted with permission from [53].

Figure 2a shows the schemes of normal SERS and G-SERS. In normal SERS (upper panel, Figure 2a), the Raman scattering of analytes located at nanogaps are significantly enhanced due to the EM coupling. In G-SERS, a one-layer graphene covers the surface of metal nanoislands (lower panel, Figure 2a), so that the active surface for SERS sensing is atomically flat, and meanwhile, sizable local EM field at the nanogaps penetrates the graphene layer and enhances the Raman scattering of analytes. The enhancement factor of G-SERS is of the same order of magnitude as, and in fact slightly larger than, normal SERS on the same type of metal nanoislands. Compared to the graphene-enhanced Raman scattering without metal [54], the clear fingerprint features of the analyte are remained, while the Raman signals are greatly enhanced due to the EM contribution.

G-SERS has also shown some unique features that make it perfect candidate for practical SERS sensing. The size of a G-SERS substrate can be easily scaled up and is only limited by the surface area of the substrate for graphene growth. The authors demonstrated a transparent, freestanding and flexible “G-SERS tape” as large as 8 × 8 cm^2^. The G-SERS tapes are highly stable and reusable. Figure 2b shows the real time and reversible detection of R6G molecules using a G-SERS tape. If placed on pure water, the G-SERS tape only produces a characteristic peak assigned to the G-band of graphene (curve I). However, if the tape is placed on an aqueous solution of 1 × 10^−5^ M R6G, the Raman peaks of R6G are clearly observed (curve II), and then disappear if the tape is moved on pure water again (curve III). Another example is given in Figure 2c, where G-SERS spectrum of CuPc adsorbed on cauliflower (soaked in 1 × 10^−5^ M CuPc in ethanol for 10 min) shows distinct Raman peaks (black curve) and the normal Raman spectrum (red curve) does not show any vibrational feature of the analyte.

### 2.3. Ultra-High-Vacuum TERS

**Figure 3.**
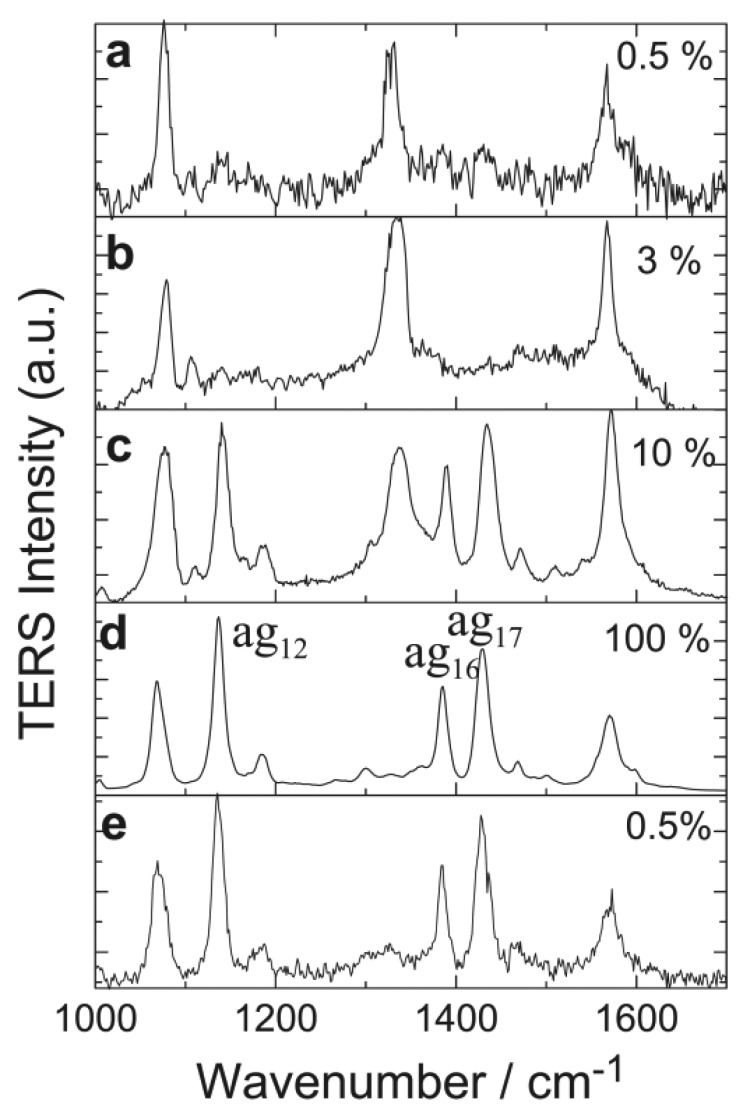
UHV-TERS spectra showing the dimerization of 4-nitrobenzenethiol (4NBT) to dimercaptoazobenzene (DMAB) molecules. Reprinted with permission from [59].

TERS utilizes either the EM enhancement of the metal tip or the EM coupling at the nanogap between the tip and the metal substrate or metal nanostructures on substrate [55–57]. In an ambient environment, TERS is affected by small molecules in the air, such as water and oxygen, so that the intrinsic spectrum of the analyte is sometimes complicated. This effect can be avoided if TERS is performed under ultra-high vacuum (UHV). From the fundamental point of view, TERS can reveal rich spectroscopic information on the molecule-metal interactions and the orientation of single molecules at even atomic resolution with scanning tunneling microscope [58], although, from the sensing aspect, TERS is not even likely applicable in practice. However, UHV-TERS is discussed herein in the sense that it indeed is able to “sense” unknown species, *i.e.*, the product of plasmon induced chemical reactions [59]. Figure 3 shows the 4-nitrobenzenethiol (4NBT) molecules are dimerized to dimercaptoazobenzene (DMAB). It is seen that the dimerization begins to occur at >3% laser power (∼60 μW/μm^2^), and becomes fully completed at 100% laser power (∼2 mW/μm^2^). The dimerization turns out to be irreversible, as the TERS spectrum remains that of DMAB (the product) if the laser power is reduced to 0.5% (∼10 μW/μm^2^). Similar chemical reactions have also been observed for other molecules. This chemical reaction is believed to be attributed to plasmon induced “hot” electrons that provide extra energy to overcome the potential barrier.

## 3. LSPR/SPP Sensing Techniques

### 3.1. Sensing Using Fano Resonances, Multipolar SPRs and Lattice Plasmon Resonances

The fact that LSPRs in metal nanostructures depend strongly on their dielectric environments enables the sensing of local refractive index using metal NPs. For chemical or biological species close to a NP, the contrast in the refractive index compared with the surrounding medium can induce a measurable shift of the LSPRs in the metal NPs, making LSPR capable of probing a nanoscale volume of species. Excellent reviews on LSPR sensing can be found in the literature [5,13]. The performance of a LSPR sensor can be characterized by the FoM, defined as the ratio of peak-shift/RIU to the peak linewidth [36]. It should be noted that other definition of FoM considering the intensity change instead of peak shift is also proposed to characterize the sensor performances [60–64]. New trends to get larger FoM are to exploit dark plasmons or lattice plasmons taking advantage of their narrower linewidths. Representative examples include sensing based on Fano resonances [65–69], multipolar SPRs [70–73] and lattice plasmon resonances [38,40,41].

**Figure 4.**
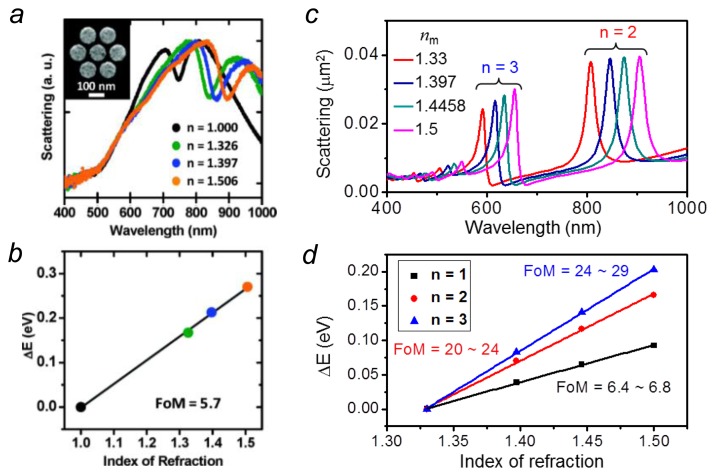
(**a**,**b**) Experimental demonstration of LSPR sensing using a gold heptamer. Reproduced with permission from [68]; (**c**,**d**) Electromagnetic calculations comparing the sensing performance using different orders (*n* = 1, 2, 3) of LSPRs on a silver nanorod. Adapted from [71].

Resulting from the coherent interference between a dark and a bright mode, Fano resonances in plasmonic NPs are sensitive to local change of the refractive index around the NPs. In addition, the narrow linewidths of the dark modes can further improve the sensing FoM. So far, LSPR sensing using Fano resonances have achieved a FoM of 3.8 for a coupled dipole-quadrupole antenna [74] and 5.7 for a gold disk heptamer [68], as shown in Figure 4a,b. Earlier experiments reported that a simple configuration—a single silver nanocube on dielectric substrate was able to produce a high FoM of 5.2 [36]. But later theoretical investigations revealed that the high FoM also attributes to a Fano resonance which comes from the substrate mediated coupling of the dark quadrupolar and bright dipolar mode in the silver nanocube [69]. At optimized conditions, this nanocube on substrate configuration can produce a high FoM ranging from 12 to 20 [69].

Compared to dipolar plasmon modes usually associating with large radiative damping, multipolar LSPRs have narrower linewidths due to the reduced radiative damping. Therefore, a higher FoM can be obtained for sensing based on multipolar LSPRs. For an individual silver nanorod, the sensing FoM can achieve 24–29 for the *n* = 3 order resonance compared to 6.4–6.8 for the dipolar one (*n* = 1) [71], as shown in Figure 4c,d. Another attractive trend toward ultrasensitive chemical or biological sensing is to use the lattice plasmons involving the diffraction coupling of the LSPRs. Sharp resonances appear in the transmission spectra of periodic metallic nanostructure, such as a NP or nanohole array [75,76]. Taking advantage of the subradiative collective modes, sensing based on these structures can show extremely high FoM about one order of magnitude higher than those in individual NPs [41,77]. For example, in a suspended nanohole array, the sensing FoM can go as high as 162 which enables a convenient identification of a monolayer of protein [41]. In Au nanorod arrays, the FoM as high as 330 was reported [38].

### 3.2. Nanowire-Based SPP Sensing

**Figure 5.**
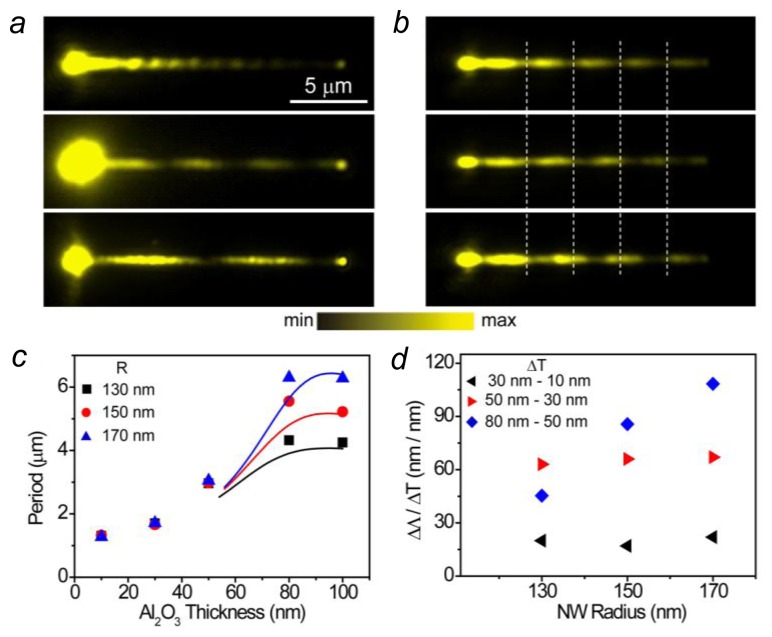
(**a**) QD emission images for a 155 nm radius NW coated with 15 nm of Al_2_O_3_ and QDs measured in air (Top), water (Middle), and oil (Bottom); (**b**) QD emission images for a 162 nm radius NW with a 50 nm Al_2_O_3_ coating measured in air (Top), and then after depositing 5 nm of Al_2_O_3_ (Middle), and finally with an additional 5 nm of Al_2_O_3_ (Bottom). The white dashed lines are visual guides to show the shift of the plasmon near-field pattern. Scale bar in (**a**) is for (**b**) as well; (**c**) The beat period as a function of Al_2_O_3_ coating thickness. The dots are experimental data, and the lines are calculated data; (**d**) The period change per nanometer of Al_2_O_3_ as a function of the nanowire radius (Adapted from [42]).

Metal NWs supporting propagating SPs have attracted attention for their potential application in nanophotonic circuits [4,78]. The properties of NW plasmons are sensitively dependent on the dielectric environment, which makes plasmonic NW a new type of structure for sensing applications [42]. When the excitation light is focused onto the end of the NW with polarization parallel to the NW, two modes can be excited. The coherent superposition of these two modes results in the plasmon beating on the NW. Figure 5a shows the near field beating patterns on a silver NW detected by the fluorescence of QDs on the NW. When the NW is measured in air, the near field period *Λ* is about 1.3 μm (top panel in Figure 5a). By immersing the NW in water, increasing the refractive index *n* of the surrounding medium from 1.00 to 1.33, the period is dramatically increased to 4.4 μm (middle panel in Figure 5a). Replacing the water by oil of refractive index 1.51 further increases the period to 7.2 μm (bottom panel in Figure 5a). Therefore, the change in period per refractive index unit (RIU) is Δ*Λ*/Δ*n* = 16 μm/RIU for the medium change from water to oil. The node shift of the *N-th* period can cumulate, leading to a sensitivity of *N* Δ*Λ*/Δ*n*. For the NW in Figure 5a, there are two periods in oil. Thus the sensitivity for the second period is 32 μm/RIU from water to oil.

Changing the local dielectric surroundings by depositing Al_2_O_3_ layer onto the NW surface can also markedly change the near field period (Figure 5b). For a Ag NW originally coated with 50 nm of Al_2_O_3_, the period of the near field distribution is increased from 2.9 μm (top panel) to 3.3 μm when 5 nm of Al_2_O_3_ is deposited (middle panel), and to 3.8 μm with an additional 5 nm of Al_2_O_3_ layer (bottom panel). On average, the period is increased 90 nm by depositing 1 nm of Al_2_O_3_ layer. The node shift of the fourth period is cumulated to 360 nm per nanometer of Al_2_O_3_. Figure 5c shows the period dependence on the Al_2_O_3_ thickness. As can be seen, the period is increased with the increase of the Al_2_O_3_ thickness at the beginning, and finally becomes saturated for thick Al_2_O_3_. The sensitivity of period to Al_2_O_3_ thickness is also increased with the Al_2_O_3_ thickness at the beginning, and the NW originally coated with 50 nm of Al_2_O_3_ shows the largest sensitivity (Figure 5d). In addition, the thicker NW is more sensitive to the change of Al_2_O_3_ thickness (blue dots in Figure 5d). The change of the local dielectric environment can be caused by the adsorption of molecules onto the NW. The plasmonic NWs thus provide a new scheme for chemical and biological sensing beyond the metal film and NP based SPRs.

**Figure 6.**
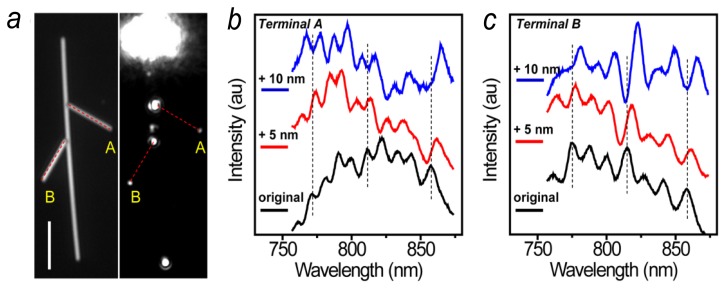
(**a**) A structure composed of three NWs was illuminated by the supercontinuum light. The length of the scale bar is 5 μm; (**b**) The emission spectra at the terminal A of the right branch wire for the original structure (black), and for 5 nm (red) and 10 nm (blue) Al_2_O_3_ layer deposited, respectively. The dashed lines are visual guides to show the shift of the peaks; (**c**) The emission spectra at the terminal B of the left branch wire (Adapted from [42]).

Besides the near field distribution period, the transmission spectra from the NW can also be used for sensing. Figure 6a shows a NW network composed of three NWs. SPs are launched from the top end of the main NW, and the transmission spectra are measured at terminals A and B, respectively. The black curves in Figure 6b,c show the spectra from the NW structure originally coated with 30 nm of Al_2_O_3_. The intensity oscillations in the spectra are due to the Fabry-Pérot (FP) resonances in the NW which can function as a FP cavity [79]. By depositing 5 nm of Al_2_O_3_, the FP resonance peaks are red-shifted as shown by the red spectra. Coating the NW with additional 5 nm of Al_2_O_3_ red-shift the spectra further (blue spectra). The results in Figure 6 not only show that the transmission spectra of NWs can be used for sensing, but also indicate that sensing can be implemented in nanophotonic network which may be used for multiplex and remote sensing applications. The dependences of spectra and near field distributions on dielectric environments provide new approaches to detect the film thickness and refractive index, and may be developed for thin-film characterizations.

For substrate-supported plasmonic NW, the propagating SPs can radiate to the substrate with the radiation angle sensitive to the NW environment. For a Ag NW on glass substrate, by depositing 1 nm of Al_2_O_3_ onto the NW, the increase of the SP radiation angle can be close to 1 degree, which provides another way to use plasmonic NW for new type sensors [43]. Moreover, plasmonic NWs provide various possibilities to combine different sensing platforms and design new sensing schemes. By using the propagating SPs on metal NWs, remote-excitation SERS sensing was realized in NW-NP composite structures as discussed above [49]. By combining metal NWs with optical fibers, optical sensing of ammonia gas in hybrid photon-plasmon all-fiber Mach-Zehnder interferometer has been demonstrated [80]. The sub-wavelength light guiding ability and the sensitive optical response to the environmental variation make plasmonic NWs versatile for new types of optical sensors.

## 4. Summary

The developments of plasmonic sensors prospect for more efficient, low-cost, stable and reusable substrates with the potential toward device applications. Despite the fact that sensing techniques based on surface-enhanced spectroscopy and SPRs have been extensively studied, the exploration of new methods that overcome existing drawbacks and improve the sensing performance is consistently pursued. The advances discussed in this review article are along this line. It is worth pointing out that, these results have showed fundamental feasibility of new sensing methods, but there is still a long way to go before these can be translated into practical sensing devices, thus stimulating further research not only in these particular systems, but also in the field of plasmonic sensors.
